# HBV HBx-Downregulated lncRNA *LINC01010* Attenuates Cell Proliferation by Interacting with Vimentin

**DOI:** 10.3390/ijms222212497

**Published:** 2021-11-19

**Authors:** Lipeng Gan, Qilin Shangguan, Fang Zhang, Xiaomei Tong, Dandan Qi, Yan Zhao, Xin Ye

**Affiliations:** 1 Key Laboratory of Pathogenic Microbiology and Immunology, Institute of Microbiology, Chinese Academy of Sciences (CAS), Beijing 100101, China; ganlipeng93@163.com (L.G.); woshinijia005@gmail.com (Q.S.); 18702996513@163.com (F.Z.); tongxm2004@hotmail.com (X.T.); qidd@im.ac.cn (D.Q.); 2 Savaid Medical School, University of Chinese Academy of Sciences, Beijing 100049, China; 3 Department of General Surgery, Strategic Support Force Medical Center, No.9 Anxiang Beili, Chaoyang District, Beijing 100101, China; ZYWQXY@sohu.com

**Keywords:** HBV, *LINC01010*, vimentin, cell proliferation

## Abstract

Hepatitis B virus (HBV) infection is closely related to hepatocellular carcinoma (HCC) development. To investigate the mechanism of HBV causing HCC, we previously analyzed the transcription of the HBV-transgenic cell line HepG2-4D14 and parental HepG2 cells and identified a subset of long noncoding RNAs (lncRNAs) differentially expressed between them. In this study, we focus on lncRNA *LINC01010*, as it is significantly downregulated in HepG2-4D14 cells and in liver tissues of HCC patients, and positively correlated with survival. We found that HBV-encoded HBx can reduce the transcription of *LINC01010*. Functional analysis showed that the overexpression of *LINC01010* inhibits proliferation, migration and invasion of HepG2 cells while the knockdown of *LINC01010* promotes these processes. By taking the approach of RNA immunoprecipitation (RIP) and mass spectrometry, we identified that *LINC01010* can interact with vimentin. Further studies demonstrated that *LINC01010* negatively affects the vimentin network extension and causes more rapid subunit exchange and lower stability of vimentin filaments. In addition, *LINC01010* can reduce the amount of insoluble vimentin within cells, which suggests that *LINC01010* interfers with vimentin polymerization. These data indicate that *LINC01010* can inhibit the assembly of vimentin filament. Thus, we revealed that HBV HBx-downregulated *LINC01010*, which suppresses cell proliferation and migration by negatively regulating the formation of vimentin filament. Taken together, *LINC01010* is a potential tumor suppressor that may restrain HBV-related HCC development.

## 1. Introduction

Liver cancer is the fourth most lethal cancer with a low five-year survival rate. Additionally, there are about 841,000 new cases and 782,000 deaths annually worldwide [1]. Hepatocellular carcinoma (HCC) is the most common primary liver cancer, accounting for approximately 80% of it [2,3]. More than 50% of HCC cases are related to hepatitis B virus (HBV) infection [4,5,6]. Chronic HBV infection leads to hepatocarcinogenesis through direct and indirect mechanisms.

HBV contributes to HCC directly via altering the genomic stability [7]. HBV DNA insertion into the telomerase reverse transcriptase (TERT) promoter activates telomerase reactivation and promotes cell immortalization [8,9]. HBV insertion targets the cyclin A gene, resulting chimeric protein and carcinogenesis [10]. HBV contributes to HCC development indirectly through HBV virus proteins. HBV-encoded HBx is a key regulatory protein that acts as a transcriptional coactivator and hijacks the host factor, playing a leading role in the development of HCC [11,12]. HBx interacts with the C terminal of p53, blocks nuclear import of p53 and impairs p53-mediated apoptosis [13]. HBx binds to CBP/P300 and enhances CREB transcriptional activity, which in turn promotes cell transformation [14]. HBx is also reported to upregulate the stability and transcriptional activity of the hypoxia-inducible factor 1-alpha (HIF1α) which leads to angiogenesis [15].

As thousands of lncRNAs have been identified, many of them are reported to play important roles in different kinds of cancers, including HCC [16,17]. LncRNA *BCRT1* stabilizes polypyrimidine tract-binding protein 3 (PTBP3) through sponging miR-1303 and promotes breast cancer progression [18]. LncRNA *Pvt1b*, which is activated by p53, suppresses the transcription of myc proto-oncogene and represses lung cancer tumorigenesis [19]. LncRNA *SATB2-AS1* enhances the transcription of SATB2 by binding to growth arrest and DNA damage-inducible protein GADD45 alpha (GADD45A) and WD repeat-containing protein 5 (WDR5), which in turn inhibits the metastasis of colorectal cancer [20]. LncRNA *HULC* promotes the phosphorylation of Y-box-binding protein 1(YB-1) through extracellular signal-regulated kinase ERK, which accelerates the translation of oncogene mRNA, eventually resulting in the progression of HCC [21]. HBx-up-regulated lncRNA *MALAT1* promotes the development and metastasis of HCC by stimulating the expression of latent transforming growth factor β-binding protein 3 (LTBP3) [22]. HBx-associated lncRNA *ATB* stimulated by transforming growth factor (TGF)-β promotes cell invasion and migration by inducing autophagy in primary liver cancer [23]. HBx-downregulated lncRNA *Dreh* can bind to vimentin and inhibit the metastasis of HCC [24].

In order to explore the role of HBV-associated lncRNAs in the progression of HCC, we performed RNA deep sequencing to compare the transcription of HBV-positive (HepG2-4D14) and HBV-negative (HepG2) cells and identified a series of differentially expressed lncRNAs [25,26]. In this study, we focus on *LINC01010*, which is significantly downregulated in HepG2-4D14. We found that the HBV HBx can down-regulate the transcription of *LINC01010*. Functional analysis showed that *LINC01010* can inhibit the proliferation, migration and invasion of HepG2 cells. Further investigation demonstrated that *LINC01010* interacts with vimentin and inhibits the cell extensions and reduces vimentin filament assembly. In conclusion, we reveal that *LINC01010* is a novel HBV-related lncRNA which negatively regulates cell proliferation, migration and invasion by interacting with vimentin. HBx down-regulates the transcription of *LINC01010* to attenuate its function, which may contribute to HCC progression.

## 2. Results

### 2.1. HBV HBx-Downregulated lncRNA LINC01010 Is Abnormally Expressed in HCC

**Figure 1:**
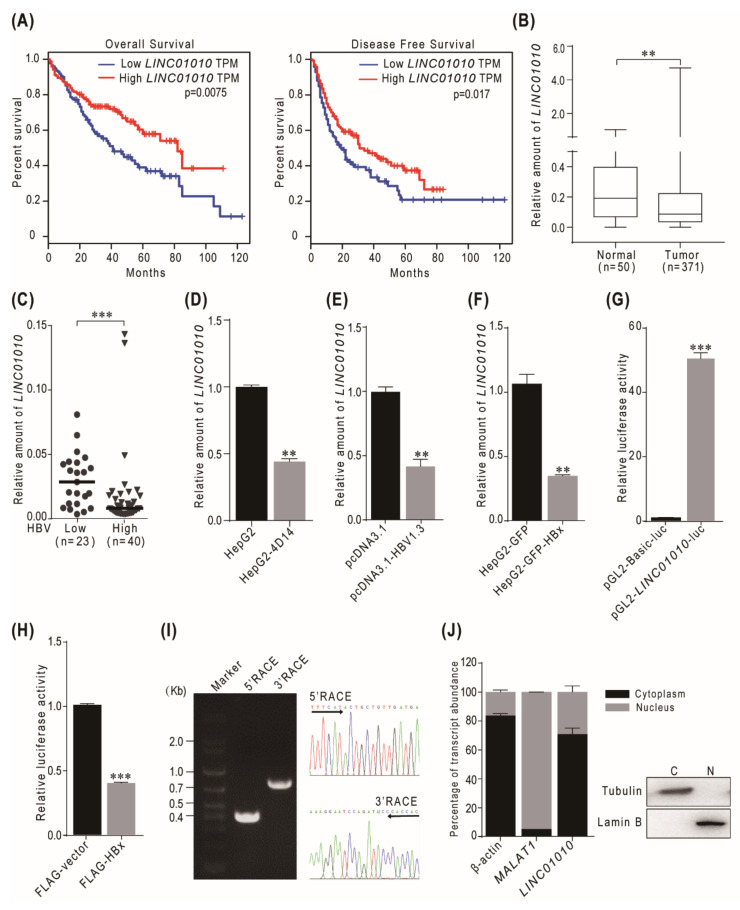
*LINC01010* is an HBV-associated lncRNA and downregulated in HCC. (**A**) Kaplan–Meier analysis of overall survival (left) and disease-free survival (right) in patients derived from the TCGA-LIHC cohort based on *LINC01010* levels. (**B**) *LINC01010* levels in HCC tumor tissues (*n* = 371) and normal tissues (*n* = 50) in TCGA-LIHC cohort. (**C**) The relative levels of *LINC01010* in 63 HCC samples were determined by real-time PCR. The samples were divided into two cohorts according to HBV copies in serum (low: *n* = 23, less than 40 copies/mL; high: *n* = 40, more than 40 copies/mL). (**D**) Relative levels of *LINC01010* in HepG2 and HepG2-4D14 cells were determined by real-time PCR and normalized with GAPDH as an internal control. (**E**) L02 cells were transfected with pcDNA3.1 or pcDNA3.1-HBV1.3 for 72 h. Total RNA was extracted, and the levels of *LINC01010* were determined by real-time PCR. (**F**) The amount of *LINC01010* in HepG2-GFP and HepG2-GFP-HBx stable cell lines was determined by real-time PCR and normalized with GAPDH as an internal control. (**G**) pGL2-*LINC01010*-luc was transfected into 293T cells, and luciferase reporter assay was performed after 48 h with pGL2-basic luciferase as control. (**H**) 293T cells were co-transfected with pGL2-*LINC01010*-luc and pCMV FLAG-HBx or pCMV FLAG-vector for 48 h. The cell lysates were collected and subjected to luciferase assays. (**I**) Total RNA was extracted from HepG2 cells and subjected to 5′ and 3′ RACE assays for full length of *LINC01010*. The RACE PCR products of *LINC01010* (left) and sequencing data (right) are shown. (**J**) The total RNA was extracted from cytoplasmic (C) and nuclear (N) fractions in HepG2 cells and subjected to real-time PCR to quantify *LINC01010* using MALAT1 and β-actin as controls. The fractionated lysates were immunoblotted with tubulin and lamin B. ** *p* < 0.01 and *** *p* < 0.001, means ± SD are shown.

In order to identify the genes affected by HBV, we previously compared the transcriptional profiles of the HBV-transgenic cell line HepG2-4D14 and its parental cell line HepG2 and identified 64 downregulated lncRNAs [25]. Among the top five downregulated genes, lncRNA *LINC01010* is the one correlated with HCC based on the data of the overall survival and disease-free survival in patients in the TCGA liver hepatocellular carcinoma (TCGA-LIHC) cohort (Figure 1A). The expression level of *LINC01010* was lower in tumor tissues than in normal tissues in the TCGA-LIHC cohort (Figure 1B). To investigate the correlation of *LINC01010* with HBV, we examined the *LINC01010* level in 63 HCC tissues. The clinicopathologic features of the patients are listed in Figure S1A. The results show that *LINC01010* was obviously lower in tumors with a high HBV virus load than in tumors with a low HBV virus load (Figure 1C). We also confirmed that *LINC01010* was downregulated in HepG2-4D14 cell lines compared to parental HepG2 cells using real-time PCR (Figure 1D). At the same time, we found that the level of *LINC01010* was lower in the cells transfected with pcDNA3.1-HBV1.3 than in the control (Figure 1E). These data demonstrate that *LINC01010* was negatively correlated with HBV infection and HCC. Next, we investigated how HBV downregulates *LINC01010*. As HBV-encoded HBx is a transcriptional regulator, we checked whether HBx regulates the expression of *LINC01010*. The data of real-time PCR show that *LINC01010* was lower in the HBx stably expressed cells than in the control cells (Figure 1F). The luciferase reporter assay demonstrated that HBx-downregulated the promoter activity of *LINC01010* (Figure 1G,H and Figure S1B,C). These results indicate that HBx negatively regulated the transcription of *LINC01010*. We performed the 5′ and 3′ RACE to confirm that the full length of *LINC01010* is 1508 nt (Figure 1I) and a cell fractionation assay to show that *LINC01010* is mainly located in the cytoplasm (Figure 1J). Previous studies demonstrated that cytoplasmic lncRNAs mainly exert their roles in modulating protein translation, protein stability or protein–protein interaction, consequently affecting multiple cellular processes [27,28], which implies that *LINC01010* may affect the function of intracellular proteins.

### 2.2. LINC01010 Attenuates the Cell Proliferation, Migration and Invasion

**Figure 2:**
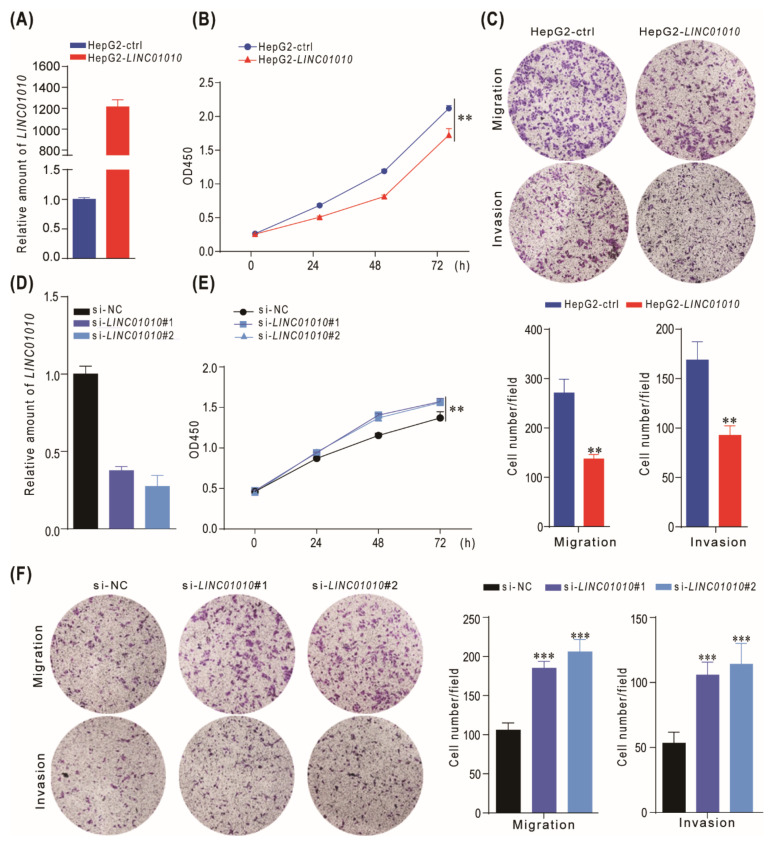
*LINC01010* inhibits HCC cell proliferation, migration and invasion. (**A**) HepG2 cells were infected with lentivirus carrying *LINC01010* or control lentivirus. The relative level of *LINC01010* was quantified by real-time PCR. (**B**) The growth curve of HepG2 overexpressing *LINC01010* or control cells were performed by CCK-8 assay. (**C**) The migration and invasion of *LINC01010*-overexpressed HepG2 or control cells were determined by transwell assay. The representative of migration and invasion assays (top panels) and the corresponding statistical results (bottom panels) are shown. A total of 4 × 10^4^ cells were used for migration assay, and 8 × 10^4^ cells were used for invasion assay. (**D**) The siRNAs targeting *LINC01010* (si-*LINC01010*#1, si-*LINC01010*#2) or control siRNA were transfected into HepG2 cells for 48 h. Then, the total RNA was extracted and subjected to real-time PCR. (**E**) HepG2 was transfected with si-RNAs of *LINC01010* and then subjected to CCK8 assay. (**F**) The migration and invasion of *LINC01010* knockdown HepG2 cells and control cells were measured by transwell assay. The representative images (left panels) and the corresponding statistical results (right panels) are shown. A total of 3 × 10^4^ cells were used for migration assay, and 6 × 10^4^ cells were used for invasion assay. ** *p* < 0.01, *** *p* < 0.001, means ± SD are shown.

To gain insight into the functional role of *LINC01010* in the HCC cell, we overexpressed *LINC01010* in HepG2 and LM3 cells (Figure 2A and Figure S2A). The growth curve demonstrated that the overexpression of *LINC01010* inhibited the proliferation of HepG2 and LM3 cells (Figure 2B and Figure S2B). Then, we further explored the effects of *LINC01010* on HCC cell migration and invasion since these are two criteria to evaluate the ability of cancer metastasis. We performed the transwell assay to determine whether *LINC01010* influenced the migration and invasion of HCC cells. These results reveal that *LINC01010* delayed the migration and invasion capabilities of HepG2 and LM3 cells (Figure 2C and Figure S2C). Then, we used siRNA to knockdown the expression of *LINC01010* (Figure 2D and Figure S2D) and performed the abovementioned experiments. The data indicate that the knockdown of *LINC01010* promoted the proliferation, migration and invasion of HepG2 and LM3 cells (Figure 2E,F and Figure S2E,F). Then, we performed the rescue experiment using the transwell and CCK8 assay. The results show that *LINC01010* can rescue the proliferation, invasion and migration phenotypes of HepG2 cells due to the knockdown of *LINC01010* (Figure S3A,B). Taken together, these results indicate that *LINC01010* inhibits the proliferation, migration and invasion of HCC cells.

### 2.3. LINC01010 Interacts with Vimentin

**Figure 3:**
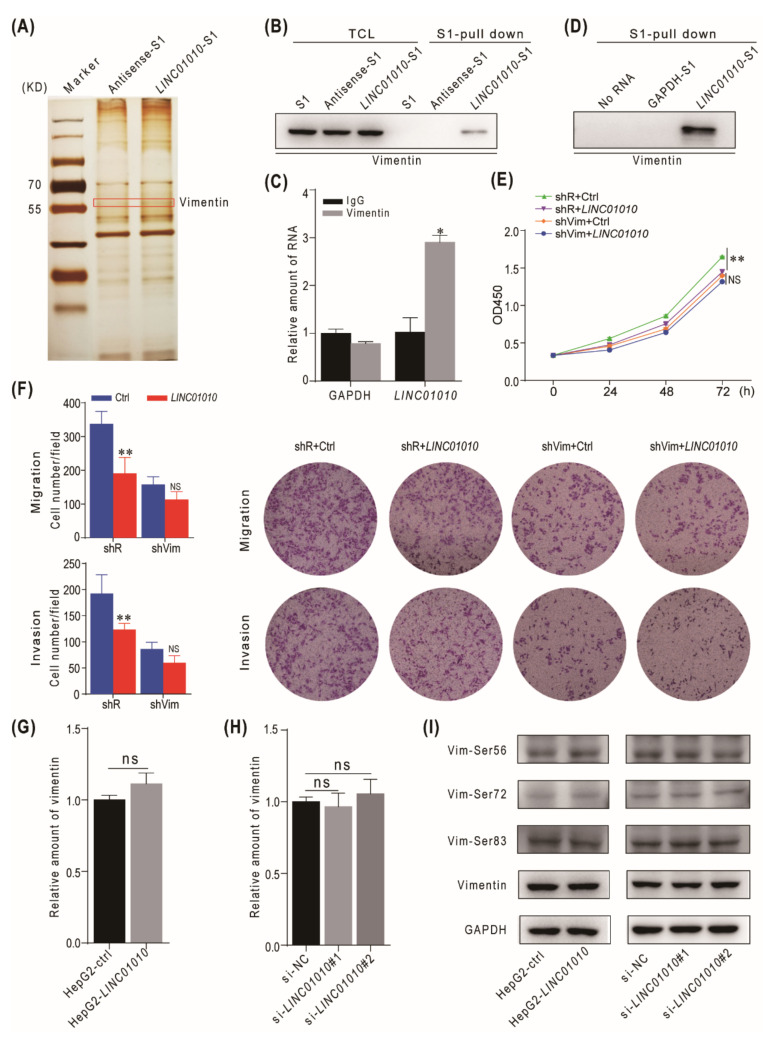
*LINC01010* interacts with vimentin. (**A**) The plasmids expressing *LINC01010*-S1 or Antisense-S1 were transfected into L02 cells. The cell lysates were harvested and subjected to the S1 pull-down assay. The pull-downed proteins were visualized using sliver staining. The band in red box was subjected to mass spectrometry. (**B**) L02 cells were transfected with *LINC01010*-S1, Antisense-S1 expression plasmids or control plasmid. The cell lysates were collected and subjected to S1 pull-down assay. The bound proteins were immunoblotted with vimentin antibody. (**C**) L02 cells were transfected with pll3.7-*LINC01010*-S1 or control plasmid for 48 h. The cell lysates were collected and subjected to immunoprecipitation with vimentin antibody or control IgG. The co-precipitated RNA was subjected to real-time PCR with GAPDH as a negative control. (**D**) S1 pull-down was performed by using the recombinant vimentin with a C-terminal His-tag and *LINC01010*-S1 transcribed in vitro or GAPDH-S1 mRNA as the control, followed by immunoblotting with vimentin antibody. (**E**) HepG2 cells were infected with lentivirus carrying shVim or shR and then transfected with *LINC01010* expression plasmid or control plasmid. Growth curves of indicated cells were measured by the CCK8 assay. (**F**) HepG2 cells were infected with lentivirus carrying shVim or shR and then were transfected with *LINC01010* expression plasmid or control plasmid. The cells were then subjected to migration and invasion transwell assays. The representative of migration and invasion assays (right panels) and the corresponding statistical results (left panels) are shown. (**G**,**H**) The relative mRNA level of vimentin in *LINC01010* overexpression (**G**) or knockdown (**H**) HepG2 cells was detected by real-time PCR, ns indicates a difference which is not significant. (**I**) These protein levels in *LINC01010*-overexpressed or knockdown HepG2 cells were detected by immunoblotting with indicated antibodies. * *p* < 0.05, ** *p* < 0.01, means ± SD are shown, ns - no significant.

In order to investigate the mechanism of *LINC01010* that inhibited the proliferation, migration and invasion of cells, we attempted to identify the proteins that interact with *LINC01010*. We transfected L02 cells with S1-tagged *LINC01010* (*LINC01010*-S1) and S1-tagged *LINC01010* antisense (Antisense-S1). The cell lysates were subjected to S1 pulldown followed by silver staining and mass spectrometry to identify the potential candidate proteins. The data show that vimentin was pulled down with *LINC01010* (Figure 3A and Figure S4A). Vimentin, a major cytoskeletal component of the intermediate filament, plays a critical role in multiple cellular processes, including cell division and stress responses, as well as tumorigenesis. We chose vimentin for further study. First, we confirmed the interaction between *LINC01010* and vimentin using the S1 pulldown assay. The data indicate that *LINC01010* can interact with vimentin both in L02 and HepG2 cells (Figure 3B and Figure S4B). To further confirm the interaction of *LINC01010* with vimentin, we transfected *LINC01010*-S1 expressing vector into L02 cells and collected the cell lysates for immunoprecipitation with vimentin antibodies, followed by real-time PCR with specific primers for *LINC01010*. The data show that vimentin can interact with *LINC01010* (Figure 3C). To determine whether *LINC01010* can interact with vimentin directly, we performed an RNA pull-down assay with C-terminal His-tag recombinant vimentin and transcribed *LINC01010*-S1 in vitro. The data demonstrate that *LINC01010* can directly interact with vimentin (Figure 3D). In order to determine that *LINC01010* exerts biological functions by interacting with vimentin, we evaluated the effects of *LINC01010* in vimentin-deficient cells. We used shRNA to knockdown the expression of vimentin and performed the CCK8 and transwell experiments. The data indicate that *LINC01010* overexpression does not further inhibit the proliferation, migration and invasion phenotypes in HepG2 cells in which vimentin has been knocked down (Figure 3E,F), which suggests that *LINC01010* plays a negative regulatory role in cell proliferation, migration and invasion through vimentin. Next, we examined whether *LINC01010* influenced the expression and stability of vimentin. As shown in Figure 3G and Figure S4C, the overexpression of *LINC01010* did not influence the mRNA and protein level of vimentin. Consistently, knocking down of *LINC01010* did not affect the mRNA and protein level of vimentin (Figure 3H and Figure S4C). As the phosphorylation of vimentin at serine 56/72/83 is critical for its reorganization [29,30,31], we examined whether *LINC01010* would affect the Ser56/72/83 phosphorylation of vimentin. The data show that *LINC01010* did not influence the level of vimentin phosphorylation at Ser56/72/83 (Figure 3I). These results show that *LINC01010* negatively regulates the proliferation, migration and invasion capabilities of HCC cells via interacting with vimentin directly but not affecting the level of vimentin and its phosphorylation status.

### 2.4. Overexpression of LINC01010 Inhibits the Network Extension and Assembly of Vimentin

**Figure 4:**
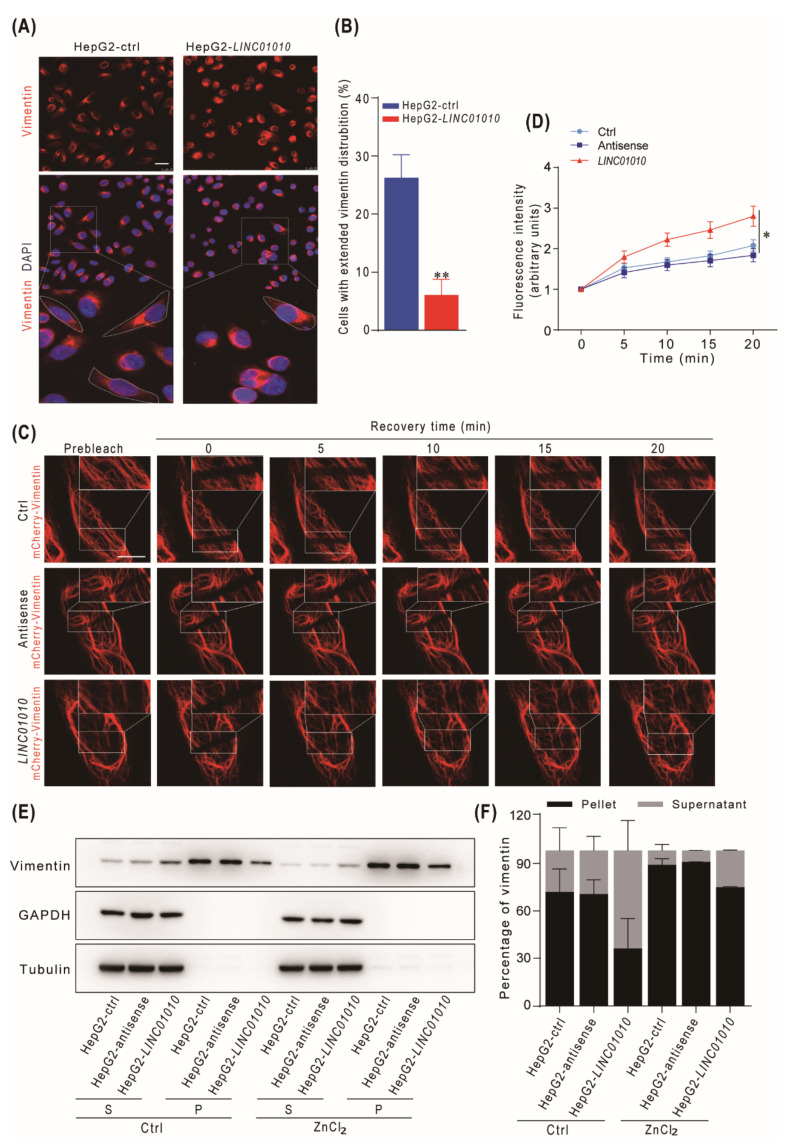
*LINC01010* reduces the stability and formation of the vimentin network. (**A**,**B**) The HepG2 cells stably expressing *LINC01010* and control cells were plated on cover glass for 4 h. The plated cells were fixed with 4% paraformaldehyde, followed by immunofluorescence assays with vimentin antibody. Red fluorescence (vimentin),DAPI staining (nuclei). The white dotted line indicates the cells with extended vimentin. Scale bars, 25 μm (**A**). The percentage of cells with extended vimentin was calculated (**B**). (**C**,**D**) HepG2 cells were co-transfected with mCherry-vimentin and *LINC01010*, antisense expression plasmids or control vector. Then, the cells were photo-bleached, and fluorescence recovery was monitored by acquiring images at time of 5, 10, 15 and 20 min after photo-bleaching with Leica SP8 confocal microscope. The white square indicates the photo-bleaching regions. Scale bars, 10 μm (**C**). The fluorescence intensity of the bleached region at the different time points after quenching was measured by ImageJ software (Ctrl, *n* = 10; antisense, *n* = 10; *LINC01010*, *n* = 10) (**D**). (**E**,**F**) HepG2-*LINC01010*, HepG2-antisense or HepG2-ctrl cells were treated with and without ZnCl_2_ (30 μM) for 12 h. Then, the cell lysates were harvested and fractionated into supernatant (S) and pellet (P) and subjected to immunoblotting analysis. The relative density of vimentin was calculated (E). The percentage of soluble and insoluble vimentin was measured (**F**). * *p* < 0.05 and ** *p* < 0.01, means ± SD are shown.

Normally, vimentin in cells have perinuclear distribution upon plating, and then extend to cell periphery accompanied with cell spreading in a time-dependent manner [32]. Vimentin-based cell extensions is a highly dynamic procedure which may be affected by the initial assembly of vimentin filament. Therefore, we examined whether *LINC01010* can affect the extension of the vimentin network in HepG2 cells. The data show that the percentage of cells with an extension of vimentin in *LINC01010*-overexpressed HepG2 cells was significantly reduced compared with that in control cells (Figure 4A,B). These data indicate that *LINC01010* inhibited the vimentin network extension. Vimentin filaments are dynamic in steady-state situations, and there is subunit exchange from dynamic. To check whether *LINC01010* influences the subunit exchange of vimentin filament, we used the FRAP to monitor HepG2 mCherry-vimentin cells, which were photo-bleached and recovered for the indicated time. The recovery time was obviously shorter in *LINC01010*-overexpressing HepG2 cells than in the control cells (Figure 4C,D). These data demonstrate that the overexpression of *LINC01010* causes more rapid vimentin subunit exchange and reduces the stability of the vimentin filaments in HepG2 cells. Vimentin monomers couples with each other to form dimers. Dimers are arranged in an antiparallel manner and comprise tetramers. Eight tetramers generate the unit length filaments, which then assemble mature filaments. During vimentin polymerization, zinc interacts with vimentin and promotes the filament assembly [32]. The mature filaments, which are assembled from soluble vimentin precursors, are insoluble bundles. To examine whether *LINC01010* affects the vimentin filament assembly, we treated the HepG2-*LINC01010*, HepG2-antisenseand control cells with ZnCl_2_ and examined the soluble and insoluble vimentin in cells. Immunoblotting analysis showed that *LINC01010* reduced the amount of insoluble vimentin in both ZnCl_2_-treated and untreated cells (Figure 4E,F), which indicates that *LINC01010* inhibited the assembly of vimentin filament. These data show that the overexpression of *LINC01010* reduces the stability of the vimentin filaments and inhibits an assembly of vimentin filaments, consequently restraining the vimentin network extension in HepG2 cells.

### 2.5. Knockdown of LINC01010 Promotes the Network Extension and Assembly of Vimentin

**Figure 5:**
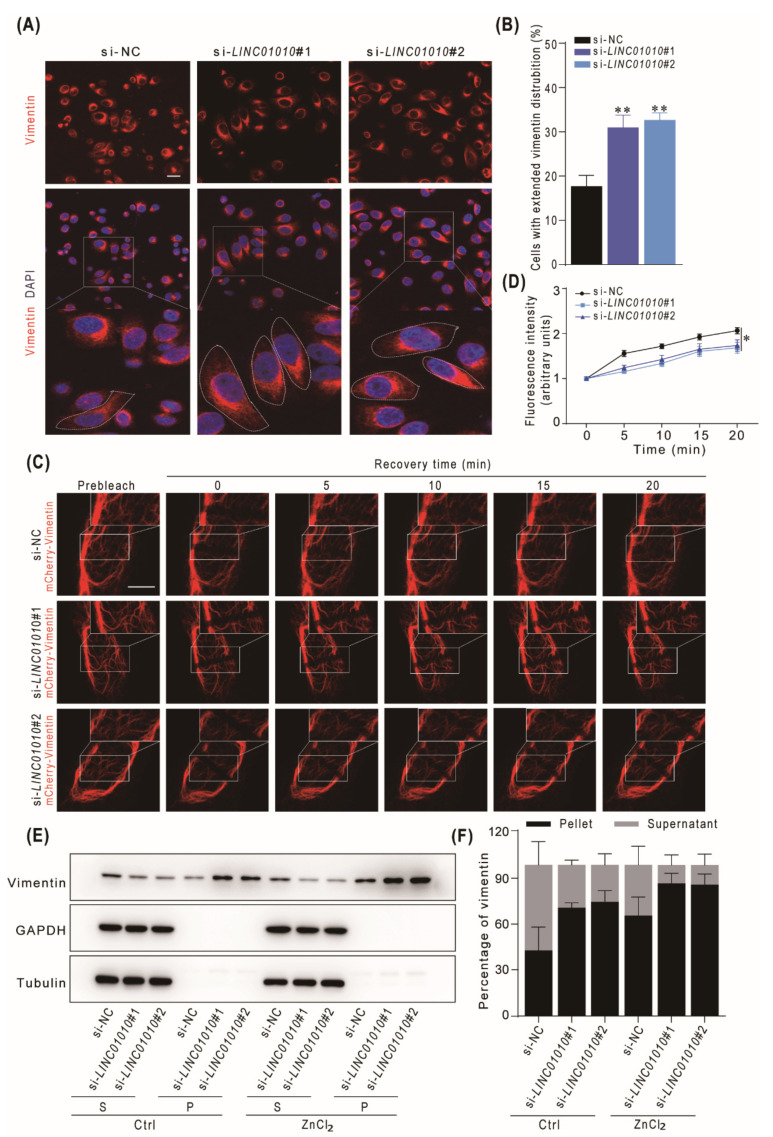
Knockdown of *LINC01010* enhances the stability and formation of vimentin network. (**A**,**B**) HepG2 cells were transfected with *LINC01010* siRNAs or control siRNA for 48 h and then plated on cover glass for 4 h and subjected to immunofluorescence assays with vimentin antibodies. Red fluorescence (vimentin),DAPI staining (nuclei). The white dotted line indicates the cells with extended vimentin. Scale bars, 25 μm (**A**). The numbers of extended cells were quantified (**B**). (**C**,**D**) HepG2 cells were transfected with *LINC01010* siRNAs or control siRNA for 24 h and then transfected with mCherry-vimentin. Then the cells were photo-bleached, and fluorescence recovery was monitored by acquiring images at time of 5, 10, 15 and 20 min after photo-bleaching with Leica SP8 confocal microscope, the white square indicates the photo-bleaching regions. Scale bars, 10 μm (**C**). The fluorescence intensity of the bleached region at the different time points after quenching was measured by ImageJ software (si-NC, *n* = 10; si-*LINC01010*#1, *n* = 10 si-*LINC01010*#2, *n* = 10) (**D**). (**E**,**F**) HepG2 cells were transfected with *LINC01010* siRNAs or control siRNA, the cells were treated with or without ZnCl_2_ (30 μM) for 12 h. Then the cell lysates were harvested and fractionated into supernatant (S) and pellet (P) and subjected to immunoblotting analysis. The relative density of vimentin was calculated (**E**). The percentage of soluble and insoluble vimentin was measured (**F**). * *p* < 0.05 and ** *p* < 0.01, means ± SD are shown.

Next, we examined the extension of the vimentin network in *LINC01010* knockdown HepG2 cells. The data show that the knockdown of *LINC01010* increased the ratio of cells with an extension of vimentin (Figure 5A,B). Then, we performed FRAP experiments and found that the fluorescence recovery time was obviously longer in *LINC01010* knockdown cells than in the control cells (Figure 5C,D). The data indicate that the knockdown of *LINC01010* enhances the stability of the vimentin filaments in HepG2 cells. We then treated the *LINC01010* knockdown HepG2 cells and control cells with ZnCl_2_ and examined the soluble and insoluble vimentin in cells by immunoblotting. The data show that knockdown of *LINC01010* increases the amount of insoluble vimentin in both ZnCl_2_-treated and untreated cells (Figure 5E,F), which demonstrated that the knockdown of *LINC01010* improves the assembly of vimentin filament.

**Figure 6:**
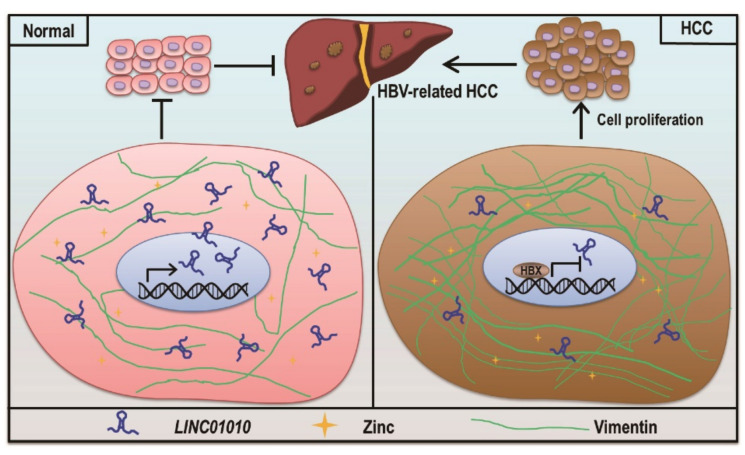
Model of the function of *LINC01010*. In normal hepatocyte cells, *LINC01010* reduces vimentin filament assembly via interacts with vimentin to attenuate cell proliferation. In HBV-infected cells, HBV-encoded HBx downregulates the transcription of *LINC01010*, which in turn promotes cell proliferation, migration and invasion, which may contribute to the development of HBV-related HCC.

In conclusion, we identified that *LINC01010* is a novel lncRNA downregulated by HBV-encoded HBx. We found that *LINC01010* inhibits proliferation, migration and invasion of HepG2 cells. We demonstrated that *LINC01010* can interact with vimentin and inhibit the vimentin network extension and interfere with the assembly of vimentin filament (Figure 6). Our study revealed that HBx reduces the level of *LINC01010* to attenuate its inhibitory effect on cell proliferation and migration, which may play role in HCC development.

## 3. Discussion

With the current development of genome and transcriptome sequencing technologies, increasingly more lncRNAs have been identified. The functional studies revealed that lncRNAs participate in many aspects of pathogenesis of various diseases [16,33,34]. It has been reported that lncRNAs are abnormally expressed in HCC and are related to the clinicopathological features and prognosis of HCC patients [35]. LncRNAs regulate gene expression at the transcriptional and post-transcriptional levels and mediate signal transduction to participate in various biological processes in the occurrence and progression of HCC, including proliferation, invasion, metastasis and apoptosis [36]. Interestingly, the *LINC01010* identified in this study can bind to the intermediate filament protein vimentin to participate in cytoskeletal rearrangement and regulate the proliferation, migration and invasion of HCC cells.

It has been reported that *LINC01010* is a novel biomarker for the diagnosis and prognosis for cancer. For example, *LINC01010* is positively correlated with the survival of neuroblastoma (NBL) patients and is a potential biomarker of NBL [37]. Cao et al. found that *LINC01010* inhibits the migration and invasion of lung cancer cells [38]. Our study indicated that *LINC01010* is an HBV-associated lncRNA, which is downregulated in HCC and positively correlated with the survival of HCC patients. Our data demonstrate that *LINC01010* may function as a tumor suppressor in HCC. It might be worth further investigation to ascertain if *LINC01010* could be used as a biomarker for other types of cancers.

HBV infection is a risk factor of HCC. HBV encodes an oncogenic HBx protein, which is a multifunctional regulator that modulates signal transduction, transcription, cell cycle progression, apoptosis and genetic stability by interacting with different host factors [39]. According to previous reports, the expression of vimentin protein is associated with HBx in HBV-related tumor tissues. The researcher showed that HBx could enhance vimentin expression to facilitate EMT in hepatoma cells [40]. Recently, it has been reported that HBx also regulates the transcription of lncRNAs. For instance, HBx-upregulated lncRNA *UCA1* promotes cell growth and tumorigenesis by recruiting histone-lysine N-methyltransferase EZH2 and suppressing p27Kip1/CDK2 signaling [41]. HBx/ERα complex downregulated *LINC01352* promotes the growth and metastasis of HCC cells through activating the Wnt/β-catenin signaling pathway [42]. HBx-downregulated lncRNA GAS5 inhibits the cell viability and invasion of hepatocellular carcinoma cell lines by activating Y-box-binding protein 1/p21 (YBX1/p21) signaling [43]. Our previous study showed that HBx-upregulated *Lnc-HUR1* can promote cell proliferation and tumorigenesis by inhibiting p53 transcriptional activity [25]. HBx-upregulated lncRNA *SAMD12-AS1* regulates cell proliferation and apoptosis by affecting the NPM1–HDM2–p53 axis [26]. In this study, we found out that HBx-downregulated *LINC01010* is involved in the cytoskeleton of cells through its interaction with vimentin. All these studies demonstrate that HBV HBx regulates the transcription of a variety of host factors. Further exploration of HBx-regulated lncRNAs will be helpful to understanding the mechanisms of how HBV promotes the development of HCC.

It is well known that vimentin is the main component of the intermediate filament family proteins, which is widely expressed in mesenchymal cells and has the function of maintaining cell integrity and resisting stress. Importantly, the expression of vimentin in different tumor cell lines is closely related to cancer cell growth, invasion and migration [44,45,46,47]. Vimentin is also identified as a marker of epithelial–mesenchymal transformation [44]. We found that *LINC01010* can interact with vimentin to inhibit the proliferation, invasion and migration of HCC cells. A previous study showed that lncRNA *Dreh* interacts with vimentin. However, how lncRNA *Dreh* affects the function of vimentin is not clear [24]. Here, we found that vimentin filament assembly is markedly affected by the expression of *LINC01010*. The overexpression of *LINC01010* can hinder cell migration through suppressing cell extension and reduce the stability of vimentin filaments. Previous studies demonstrated that zinc can regulate the polymerization of vimentin filaments [32]. Our results show that *LINC01010* affects vimentin both in the presence and absence of zinc, with the extent of inhibition being higher in the absence of zinc than in its presence, suggesting that zinc could protect vimentin from the inhibition caused by *LINC01010*.

It has been reported that vimentin could be a potential molecular target for cancer therapy, which opens up a new path for the development of promising therapeutic drugs [44]. At present, there is anticancer chemical compound target vimentin. Burikhanov R. et al. found that Arylquin 1 can bind to vimentin, which displaces Par-4 from vimentin for secretion and triggers the apoptosis of diverse cancer cells [48]; Zamay TN et al. developed a DNA aptamer NAS-24 which targets vimentin and induces apoptosis of mouse ascites adenocarcinoma cells both in vitro and in vivo [49]; researchers found that dietary silibinin inhibited the growth of prostate tumors and suppressed tumor progression from prostatic neoplasia to adenocarcinoma in transgenic adenocarcinoma of the mouse prostate (TRAMP) model, and interestingly, they observed that silibinin decreased level of vimentin in plasma [50]; withaferin-A can result in the aggregation of vimentin and induce apoptosis [51]; and FiVe1 can irreversibly inhibit the growth of mesenchymal-transformed cancer cells by binding the vimentin during mitosis [52]. These data indicate that vimentin is a promising anticancer drug target. The *LINC01010* can inhibit the proliferation and migration of HCC cells by interacting with vimentin, suggesting that *LINC01010* may function as a tumor suppressor and a potential target for treatment. Therefore, if we could find the molecule(s) to enhance the interaction between *LINC01010* and vimentin, they might be drug candidates for treatment of HBV-related HCC or even other cancers.

In summary, we identified that *LINC01010* is a novel HBV-related lncRNA which is downregulated by HBV-encoded HBx. We found *LINC01010* can inhibit the proliferation, migration and invasion of HCC cells by interacting with vimentin and consequently influencing the formation of the cytoskeleton. Our results suggest that the downregulation of *LINC01010* may play an important role in the oncogenesis and metastasis of HBV-related HCC.

## 4. Materials and Methods

### 4.1. Plasmids and Antibodies

The full-length of *LINC01010* or *LINC01010*-S1-tagged (S1 sequence 5′-ACCGACC AGAATCATGCAAGTGCGTAAGATAGTCGCGGGCCGGG-3′, an RNA aptamer which binds the streptavidin) genes were cloned into the lentiviral vector pLentilox3.7 to generate pll3.7-*LINC01010* and pll3.7-*LINC01010*-S1, respectively. The mCherry-Vimentin-7 was a gift from Michael Davidson (Addgene plasmid#55156) [53]. The promoter region from –952 to –16 nt in the human *LINC01010* gene was subcloned into the pGL2-basic vector to construct a reporter named pGL2-*LINC01010*-luc. The following antibodies were purchased from the indicated companies: rabbit anti-vimentin antibody (Cell Signaling Technology, Danvers, MA, USA), rabbit anti-Phospho-vimentin (Ser56) antibody (Affinity Biosciences, Cincinnati, OH, USA), rabbit anti-Phospho-vimentin (Ser83) antibody (Cell Signaling Technology, Danvers, MA, USA), rabbit anti-Phospho-vimentin (Ser72) antibody (ZEN-BIOSCIENCE, Chengdu, China), mouse anti-GAPDH antibody (Santa Cruz Biotechnology, Santa Cruz, CA, USA), mouse anti-tubulin antibody (Santa Cruz Biotechnology, Santa Cruz, CA, USA), mouse anti-lamin B antibody (Santa Cruz Biotechnology, Santa Cruz, CA, USA), horseradish peroxidase HRP-conjugated secondary antibodies (The Jackson Laboratory, Bar Harbor, ME, USA).

### 4.2. Cell Lines and Cell Culture

The human hepatoma cell line HepG2, human embryonic kidney cell line 293T and human normal liver cell line L02 were purchased from the American Type Culture Collection (ATCC, Manassas, VA, USA). The human hepatoma cell line LM3 was kindly provided by Chunping Cui (Beijing Institute of Lifeomics, Beijing, China). The HepG2-4D14 cell lines, derived from HepG2 cells by integrating full-length HBV genome in the cellular genome, were kindly provided by Dongping Xu at the 302 Hospital of People’s Liberation Army of China. The cells were cultured in Dulbecco’s Modified Eagle Medium (DMEM; Invitrogen, Carlsbad, CA, USA) with 10% (*v*/*v*) fetal bovine serum (FBS, Pan Biotech, Adenbach, Germany) and maintained in a 37 °C humidified incubator with 5% CO_2_. The 293T cells were transfected with pll3.7-*LINC01010* and the lentiviral packaging plasmids for 48 h. Then the virus particles in the supernatant were harvested to infect HepG2 cells for 12 h. Stable *LINC01010*-overexpressing cells were sorted by fluorescence-activated cell sorting (FACS) using the FACS Calibur flow cytometer instrument (BD Biosciences, San Jose, CA, USA).

### 4.3. Tumor Samples

The liver tissues of 63 patients with HCC were collected at the 302 Hospital of People’s Liberation Army of China. Patients were divided into two cohorts based on the suggestion of the physician who provided the clinical sample. All patients participating in the study signed a written informed consent document. Patient samples were assigned arbitrary identification numbers based on the order of enrollment. The study was performed according to the rules described by the Ethics Procedures and Guidelines of the People’s Republic of China and were approved by the Ethics Committee of the 302 Hospital of People’s Liberation Army of China.

### 4.4. RNA Interference

Cells were transfected with *LINC01010*-specific siRNAs or negative-control siRNA using siRNA-Mate (GenePharma Technology, Shanghai, China) according to the manufacturer’s instructions. The sequences of the *LINC01010*-siRNAs were as follows: si-*LINC01010*#1, GCGACGACGUUGAAUUCUATT; si-*LINC01010*#2, GCUUUAAGAUCCAGAGAUUTT.

### 4.5. Rapid Amplification of cDNA Ends (RACE)

To obtain the *LINC01010* full-length cDNA, the ends were amplified by 5′- and 3′-RACE using the SMARTerTM RACE cDNA Amplification Kit following the instructions of the manufacturer (Clontech, Mountain View, CA, USA). The following *LINC01010* specific primers were used: 5′RACE primer 5′-GCTTGCCTATTACCTTG GCCACACGG-3′, 3′RACE primer 5′-CTGAGGAATCGGGGAAAGAATGGAGCT-3′.

### 4.6. Luciferase Reporter Assay

Cells were cotransfected with the pGL2-*LINC01010*-luc and pCMV FLAG-HBx or control plasmid, and with pRL-TK as an internal control for 48 h. Then, the cell lysates were harvested for luciferase assay using Dual-Luciferase^®^ Reporter Assay System (Promega, Madison, WI, USA) according to the manufacturer’s instructions. Relative luciferase activity was determined by normalizing firefly luciferase activity to Renilla luciferase activity.

### 4.7. Quantitative RT-PCR (qRT-PCR)

Total RNA was isolated from tissue specimens or cell lines using TRIzol reagent (Invitrogen, Carlsbad, CA, USA). The RNA was then reverse transcribed into cDNA using TranScript One-Step gDNA Removal and cDNA Synthesis SuperMix (TransGen Biotech, Beijing, China). qRT-PCR was carried out with the following primers: *LINC01010* forward 5′-GCTGGAGCACACAAATAGCTACA-3′ and reverse 5′-CCTTGGCTTGCC TATTACCTTG-3′, vimentin forward 5′-AGTCCACTGAGTACCGGAGAC-3′ and reverse 5′-CA TTTCACGCATCTGGCGTTC-3′, GAPDH forward 5′-TCAAGAAGGTGGTGAAG CAG-3′ and reverse 5′-GAGGGGAGATTCAGTGTGGT-3′, β-actin forward 5′-GGAT CAGCAAGCAGGAGTATG-3′ and reverse 5′-AGAAAGGGTGTAACGCAACTAA-3′, MALAT1 forward 5′-GACGGAGGTTGAGATGAAGC-3′ and reverse 5′-ATTCGGGG CTCTGTAGTCCT-3′.

### 4.8. Immunoblotting

The cells were lysed with lysis buffer (20 mM Hepes, pH 7.5; 150 mM NaCl; 1 mM EDTA; 1% (*v*/*v*) Triton X-100; 10% (*v*/*v*) glycerol; proteinase inhibitor cocktail) on ice for 30 min. Cell lysates were harvested and subjected to immunoblotting with indicated primary antibodies followed by a secondary antibody conjugated with HRP.

### 4.9. Cell Growth Assay

Cell proliferation was assessed by Cell Counting Kit-8 (CCK-8) assay for the determination of cell viability (Dojindo Molecular Technologies, Kumamoto, Japan). Cells were seeded in 96-well plates with 5000 cells per well and cultured in DMEM with 1% (*v*/*v*) serum. Then, CCK-8 solution was added at 0 h, 24 h, 48 h and 72 h after plating. The absorbance at 450 nm of each well was measured with a microplate reader.

### 4.10. Migration and Invasion Assays

In vitro migration and invasion of HepG2 and LM3 cells were measured by transwell assays. Transwell assays were used to test cell migration (without matrigel in Millicell chambers) and invasion (with matrigel in Millicell chambers). Cells were detached by trypsin and then resuspended in serum-free medium. Cells were placed in the upper transwell chambers and maintained at 37 °C for 24 h. Cells were fixed with ethanol and stained with 0.1% crystal violet.

### 4.11. S1 Pull-Down

*LINC01010*-S1-expressing cells or control cells were UV cross-linked and lysed with lysis buffer (10 mM Tris-HCl, pH 7.5; 10 mM NaCl; 10 mM EDTA; 0.5% (*v*/*v*) Triton X-100; 1 mM PMSF, 1 mM DTT) containing protease inhibitor and RNase inhibitor on ice for 20 min. The cell lysates were incubated with prewashed streptavidin T1 magnetic beads (Invitrogen, Carlsbad, CA, USA) at 4 °C for 4 h. Then, the magnetic T1 beads were washed 5 times and boiled in the 2 × SDS loading buffer. The bound proteins were subjected to immunoblotting.

### 4.12. RNA Immunoprecipitation (RIP)

Cells were harvested and were washed with PBS. Then, cells were cross-linked by UV and lysed with RIP lysis buffer containing protease inhibitor (Roche, Basel, Switzerland) and RNase inhibitor (Thermo Fisher Scientific, Waltham, MA, USA). The cell lysates were incubated with 2 μg anti-vimentin antibody or mouse IgG antibody and 20 μL protein A agarose beads (Pierce, Rockford, IL, USA) for 4 h at 4 °C. The beads were washed with RIP lysis buffer, and the bound RNA was extracted from the beads and subjected to qRT-PCR to determine the amount of *LINC01010*.

### 4.13. In Vitro RNA Pull-Down Assay

S1-labeled RNAs were transcribed in vitro using the MEGAscriptTM T7 Transcription Kit (Invitrogen, Carlsbad, CA, USA). The RNA was heated to 90 °C for 2 min and put on ice for 2 min followed by setting at room temperature for 20 min to allow proper RNA secondary structure formation. The recombinant vimentin with a C-terminal His-tag was purchased from Sino Biological Inc. (Cat: 10028-H08B). In vitro-transcribed S1-labeled RNA was incubated with vimentin protein in binding buffer (140 mM NaCl; 0.5% (*v*/*v*) NP40; 50 mM Tris, pH 8.0; 1 mM EDTA; 1 mM PMSF; proteinase inhibitor cocktail and RNase inhibitor) at 4 °C overnight. Then the prewashed streptavidin T1 magnetic beads were added and incubated at 4 °C for 4 h. The beads were washed, and the bound proteins were dissolved in SDS loading buffer and subjected to immunoblotting with vimentin antibody.

### 4.14. Cell Extension Formation and Immunofluorescence Assays

For estimating the formation of cell extensions that are involve in cell intermediate fiber reconstruction, the cells were plated on fibronectin-coated glass at 50% to 70% confluency. After 4 h, the cells were fixed in 4% (*w*/*v*) paraformaldehyde for 15 min and permeabilized with 0.5% (*v*/*v*) Triton X-100 for 10 min. The cells were then incubated with rabbit anti-human vimentin antibody at room temperature for 1.5 h, followed by incubating with rhodamine-conjugated goat anti-rabbit IgG at room temperature for 1 h. The cells then were stained with DAPI and observed and photographed under confocal microscope.

### 4.15. Fluorescence Recovery after Photobleaching (FRAP)

The cells were plated on 35 mm glass bottom dishes at 50% confluency for 18 h and transfected with pCMV mCherry-vimentin plasmid. Then the cells were photo-bleached, and fluorescence recovery was monitored by acquiring images 5, 10, 15 and 20 min after photo-bleaching with Leica SP8 confocal microscope. The Leica SP8 confocal microscope equipped with a 37 °C humidified chamber with 5% CO_2_ was used. The fluorescence intensity of bleached region at the different time points after quenching was measured by ImageJ software.

### 4.16. Vimentin Solubility Assay

Vimentin solubility assay was performed according to the published procedures [54]. The cells were treated with 30 μM ZnCl_2_ for 12 h and lysed with lysis buffer (50 mM PIPES, 50 mM NaCl, 5% (*v*/*v*) glycerol, 0.1% (*v*/*v*) NP-40, 0.1% (*v*/*v*) Triton X-100, 0.1% (*v*/*v*) Tween20, 5 mM NaF, 2 mM sodium orthovanadate, 10 mM β-glycerophosphate) with protease inhibitor for 1.5 min. The cell lysates were centrifugated at 10,000× *g* for 10 min. Both supernatants and pellets were collected. The pellets were dissolved in sample loading buffer without bromophenol blue. The proteins were subjected to immunoblotting with anti-vimentin antibody. The ratio of soluble and insoluble vimentin was quantified by Image J.

### 4.17. Statistical Analysis

Statistical analysis was performed using GraphPad Prism (version 7.0) software unless otherwise indicated. Student’s t-test or Mann–Whitney U tests were used to analyze the data. Values are presented as means ± standard deviation (SD). *p <* 0.05 was deemed to be significant.

## Data Availability

This study uses public database for analysis and this data can be found in https://portal.gdc.cancer.gov/, access date: 19 November 2021.

## Supplementary Materials

The following are available online at https://www.mdpi.com/article/10.3390/ijms222212497/s1.
