# Supplementary material for: HBV HBx-Downregulated lncRNA LINC01010 Attenuates Cell Proliferation by Interacting with Vimentin
Source: Int J Mol Sci. 2021 Nov 19;22(22):12497. doi: 10.3390/ijms222212497 (PMC8620790; doi:10.3390/ijms222212497)
Supplement: Supplementary file 1 [file ijms-22-12497-s001.zip › ijms-1437522-supplementary/supplementary files/Supplemental Figure 1.pdf]

Figure S1. HBx downregulates the promoter activity of *LINC01010* in HepG2 cells.

(A) The clinicopathologic features of the patients

Features	Number of patients
**Age(year)**	
≤50	26
>50	37
**Gender**	
Male	45
Female	18
**HBsAg***	
Positive	33
Nenative	30
**TNM Stage**	
I	5
II	12
III	39
IV	1
Unknown	6

* HBsAg Hepatitis B virus surface antigen

(B)

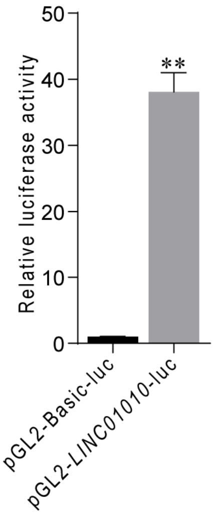

(C)

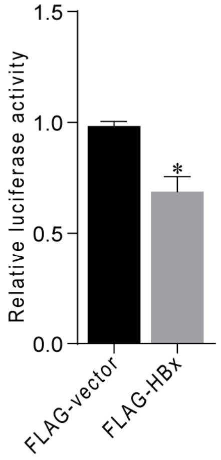

(A) Clinical information of HCC tissue samples. (B) pGL2-*LINC01010*-luc was transfected into HepG2 cells, and luciferase reporter assay was performed after 48 h with pGL2-basic luciferase as control. (C) HepG2 cells were co-transfected with pGL2-LINC01010-luc and pCMV FLAG-HBx or pCMV FLAG-vector for 48 h. The cell lysates were collected and subjected to luciferase assays.
