# Supplementary material for: HBV HBx-Downregulated lncRNA LINC01010 Attenuates Cell Proliferation by Interacting with Vimentin
Source: Int J Mol Sci. 2021 Nov 19;22(22):12497. doi: 10.3390/ijms222212497 (PMC8620790; doi:10.3390/ijms222212497)
Supplement: Supplementary file 1 [file ijms-22-12497-s001.zip › ijms-1437522-supplementary/supplementary files/Supplemental Figure 2.pdf]

Figure S2. ***LINC01010*** **inhibits LM3 cell proliferation, migration and invasion.**

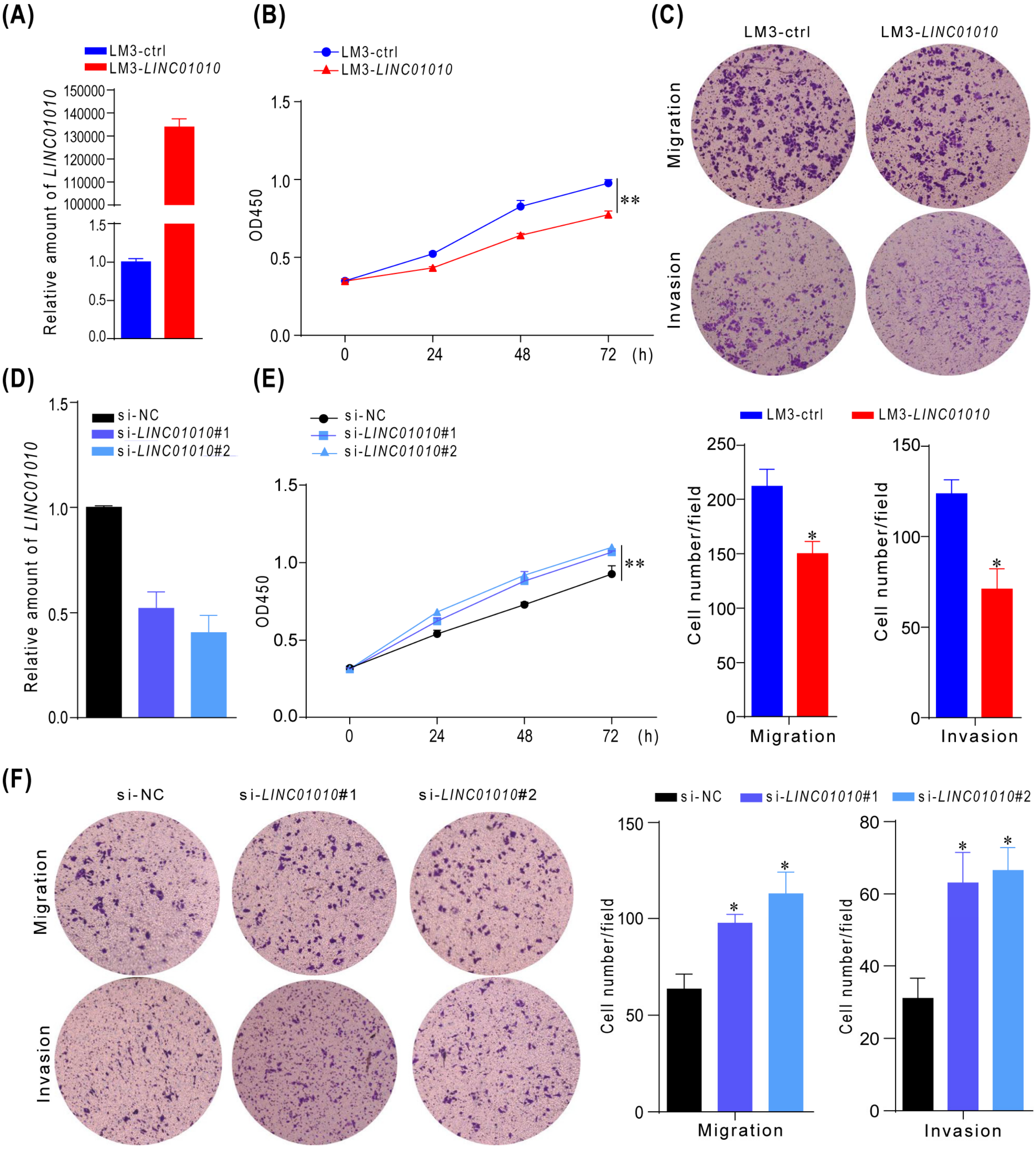

(A) pll3.7-*LINC01010* was transfected into LM3 cells. The relative level of *LINC01010* was quantified by real-time PCR. (B) The growth curve of LM3 overexpressing LINC01010 or control cells were performed by CCK-8 assay. (C) The migration and invasion of *LINC01010* overexpressed LM3 or control cells were determined by transwell assay. The representative of migration and invasion assays (top panels) and the corresponding statistical results (bottom panels) are shown. (D) The siRNAs targeting LINC01010 (si-*LINC01010*#1, si-*LINC01010*#2) or control siRNA were transfected into LM3 cells for 48 h. Then the total RNA was extracted and subjected to real-time PCR. (E) LM3 was transfected with si-RNAs of *LINC01010* and then subjected to CCK8 assay. (F) The migration and invasion of *LINC01010* knockdown LM3 cells and control cells were measured by

transwell assay. The representative images (left panels) and the corresponding statistical results (right panels) are shown. *P<0.05, **P<0.01, means ± SD are shown.
