# Supplementary material for: HBV HBx-Downregulated lncRNA LINC01010 Attenuates Cell Proliferation by Interacting with Vimentin
Source: Int J Mol Sci. 2021 Nov 19;22(22):12497. doi: 10.3390/ijms222212497 (PMC8620790; doi:10.3390/ijms222212497)
Supplement: Supplementary file 1 [file ijms-22-12497-s001.zip › ijms-1437522-supplementary/supplementary files/Supplemental Figure 3.pdf]

Figure S3. *LINC01010* inhibits the proliferation, invasion and migration of HepG2 cells.

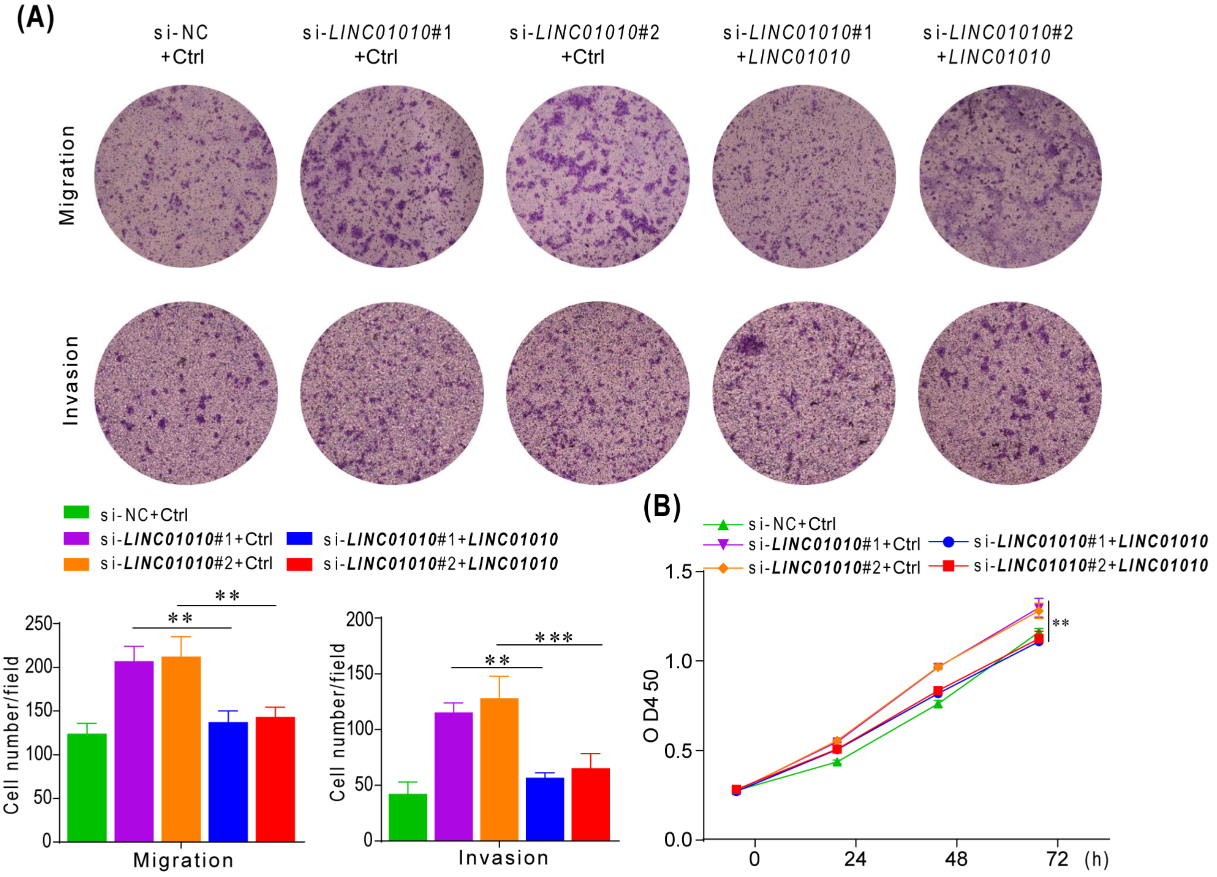

(A) HepG2 cells were transfected with si-RNAs of *LINC01010* or control siRNA and then were transfected with *LINC01010* expression plasmid or control plasmid. The cells were then subjected to migration and invasion transwell assays. The representative of migration and invasion assays (top panels) and the corresponding statistical results (bottom panels) are shown. (B) HepG2 cells were transfected with si-RNAs of *LINC01010* or control siRNA and then were transfected with *LINC01010* expression plasmid or control plasmid. Growth curves of indicated cells were measured by the CCK8 assay. ** $p < 0.01$ and *** $p < 0.001$, means ± SD are shown.
