# Supplementary material for: HBV HBx-Downregulated lncRNA LINC01010 Attenuates Cell Proliferation by Interacting with Vimentin
Source: Int J Mol Sci. 2021 Nov 19;22(22):12497. doi: 10.3390/ijms222212497 (PMC8620790; doi:10.3390/ijms222212497)
Supplement: Supplementary file 1 [file ijms-22-12497-s001.zip › ijms-1437522-supplementary/supplementary files/Supplemental Figure 4.pdf]

Figure S4. *LINC01010* interacts with the vimentin.

(A)

The bound proteins identified by mass spectrometry

Protein	Peptide	Score	Expect
Vimentin	FADLSEAANR	49.43	0.00016
Drebrin	LSSPVLHR	31.17	0.0036
DDX5	APILIATDVASR	28.97	0.0087
RPL8	AVVGVVAGGGR	28.95	0.009
Myosin-10	IVFQEFR	27.98	0.016
ACC1	LGTPELSTAER	27.3	0.033
RPL4	AAAAAAALQAK	25.65	0.029
ALPK1	QARISVNSGKLLK	20.34	0.034

(B)

(C)

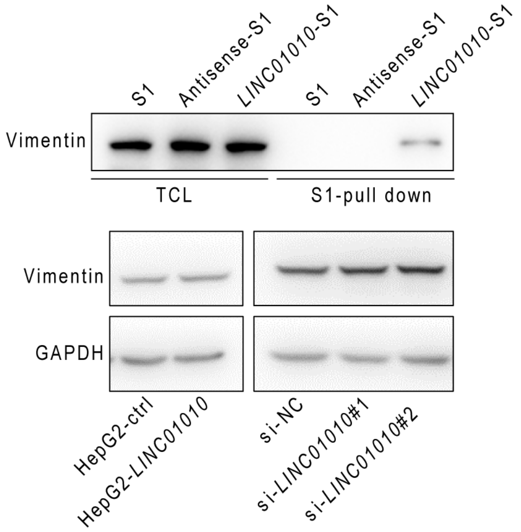

(A) The cell lysates from HepG2-*LINC01010*-S1 and HepG2-Antisense-S1 cells were subjected to S1 pull-down. The bound proteins were resolved in SDS-PAGE and subjected to mass spectrometry. The identified proteins were listed. (B) HepG2 cells were infected with a lentivirus carrying *LINC01010*-S1, Antisense-S1 or control virus. The cell lysates were collected and subjected to S1 pull-down assay. The bound proteins were immunoblotted with vimentin antibody. (C) The protein levels of vimentin in *LINC01010* overexpression or knockdowned HepG2 cells were determined by immunoblotting.
